# The Feline calicivirus capsid protein VP1 is a client of the molecular chaperone Hsp90

**DOI:** 10.1099/jgv.0.002030

**Published:** 2024-10-07

**Authors:** Carolina Pérez-Ibáñez, Yoatzin Peñaflor-Téllez, Carlos Emilio Miguel Rodríguez, Ana Lorena Gutiérrez Escolano

**Affiliations:** 1 Departamento de Infectómica y Patogénesis Molecular, Centro de Investigación y de Estudios Avanzados del Instituto Politécnico Nacional, Mexico City, Mexico

**Keywords:** autophagy, FCV, Feline calicivirus, Hsp90, proteasome, VP1

## Abstract

Feline calicivirus (FCV) icosahedral viral capsids are composed of dozens of structural subunits that rely on cellular chaperones to self-assemble in an orderly fashion. Here, we report that the heat shock protein 90 (Hsp90) inhibition significantly reduced FCV particle production, suggesting a role in the replicative cycle. We found that Hsp90 inhibition was not related to the synthesis or stability of the early proteins that translate from the gRNA nor to the minor capsid protein VP2 but with a reduction in the major capsid protein VP1 levels, both translated late in infection from the subgenomic RNAs. Reduction in VP1 levels was observed despite an augment of the leader of the capsid (LC)–VP1 precursor levels, from which the LC and VP1 proteins are produced after proteolytic processing by NS6/7. The direct interaction of VP1 with Hsp90 was observed in infected cells. These results suggest that upon release from the polyprotein precursor, VP1 becomes a client of Hsp90 and that this interaction is required for an efficient FCV replicative cycle.

## Introduction

The family *Caliciviridae* comprises non-enveloped viruses that enclose a genome that consists of a single positive-stranded RNA molecule organized into two to four ORFs. Their non-structural and structural proteins are encoded in different ORFs, and both major (viral protein 1 or VP1) and minor (viral protein 2 or VP2) capsid proteins are translated from a subgenomic RNA (sgRNA) [1,3]. There are currently 11 genera classified within the family *Caliciviridae*: of them, members of 2 genera infect birds (*Bavovirus* and *Nacovirus*), 2 infect fish (*Minovirus* and *Salovirus*), and 7 contain viruses that infect mammals (*Lagovirus*, *Norovirus*, *Nebovirus*, *Recovirus*, *Sapovirus*, *Valovirus* and *Vesivirus*) [1]. Feline calicivirus (FCV) belongs to the genus *Vesivirus* and infects both wild and domestic cats, causing predominantly upper respiratory symptoms; it was first isolated in 1957 and has been an excellent model for studying calicivirus biology since it is one of the few members of the family that is readily cultured *in vitro* and has several reverse genetic systems, and its functional receptor, the junctional adhesion molecule 1, has been identified [4,5].

The FCV genome is organized into three ORFs: ORF1 gives place to a polyprotein that is cleaved into six non-structural proteins, from NS1 to NS6/7 [6], while ORF2 and 3 encode for the structural proteins late during infection. ORF2 encodes for the major capsid protein VP1, of which 180 copies are present in each viral particle [7]. This protein is initially translated as a precursor that is processed by the viral protease to give rise to the mature VP1 and to a 14 kDa protein, named leader of the capsid (LC), associated with the establishment of the cytopathic effect typically observed during *in vitro* infection and recently reported as a viroporin responsible for apoptosis induction in a virus-free system [8,10]. ORF3 encodes for VP2, the minor capsid protein, which is present in the virion in a much lesser amount (approximately ten copies per virion) that was first reported to be essential for the production of infectious virus [2] and further characterized as responsible for the viral genome release to the cell cytoplasm after virus entry [11].

Viral proteins, because of their complexity, require cellular chaperones to achieve proper conformation and become biologically active. Over time, it has become evident that the need for these cellular proteins goes far beyond just securing the proper folding of viral proteins, since their interaction can modulate subcellular localizations or change their activity and interactions with other cellular proteins involved in different pathways, including the antiviral immune response [12].

Heat shock protein 90 (Hsp90) is a key member of the proteostasis machinery and one of the most abundantly expressed proteins in the cell, accounting for 1–2% of total cellular proteins, and under stress conditions, it can increase up to 4–6% [13]. The Hp90 system is one of the most studied in the context of viral infections [14]. Previous reports identified the requirement of Hsp90 for the successful replication of the murine norovirus (MNV), another member of the family *Caliciviridae* whose major capsid protein requires Hsp90 activity for its stability and is required for virus replication [15].

Here, we report that Hsp90 plays a crucial role in the efficient replication of FCV. We have demonstrated that, similar to the VP1 capsid proteins from MNV and a human norovirus (HuNoV), FCV VP1 is also an Hsp90 client protein. This finding opens up new avenues for understanding the molecular mechanisms of FCV replication and potentially developing novel antiviral strategies.

## Methods

### Cell cultures and viral infections

Crandell–Reese feline kidney (CrFK) cells [16], obtained from the American Type Culture Collection (Rockville, MD), were grown in Advance Eagle’s Minimal Essential Medium (MEM), with 2 mM l-glutamine, 1.0 mM sodium pyruvate, 0.1 mM nonessential amino acids and 1.5 g l^–1^ sodium bicarbonate. The medium was supplemented with 7% FBS, 5000 U of penicillin and 5 µg ml^−1^ of streptomycin. Cells were grown in a 5% CO_2_ incubator at 37 °C [17]. The FCV Urbana (URB) strain used in this study was obtained from a reverse genetic system using the pQ14 infectious clone (kindly provided by Dr. K. Green) [18]. Virus titres were determined by plaque assay as previously described [19]. A multiplicity of infection (MOI) of 5 was used in all experiments to achieve a synchronous infection [20]. Immediately before infection, CrFK cell monolayers at a confluency of 80% were washed with prewarmed 1× PBS, pH 7.4 [137 mM sodium chloride, 2.7 mM potassium chloride, 10 mM disodium hydrogen phosphate and 1.8 mM potassium dihydrogen phosphate (J.T.Backer)] [21]; viral inoculum was added in MEM without FBS, and adsorption was allowed for 1 h at 37 °C, with gentle shaking every 15 min. Afterwards, the viral inoculum was removed, and the monolayer was washed with prewarmed 1× PBS before adding MEM supplemented with 2% FBS. Depending on the experimental conditions, cells were treated with geldanamycin (GA) (Santa Cruz Biotechnology), chloroquine (CQ) (Sigma-Aldrich), carbobenzoxy-l-leucyl-l-leucyl-l-leucinal (MG132) (Sigma-Aldrich) or17-dimethylaminoethylamino-17-demethoxygeldanamycin (17-DMAG) (Merck Millipore), alone or combined at the indicated concentrations, or the drug vehicles as controls, added to the maintenance media.

### Reagents and cell treatments

A stock solution of 1 mM GA in DMSO (Sigma-Aldrich) was prepared and stored in aliquots of 10 µl at −20 °C. For each experiment, an aliquot was diluted with DMSO to a working concentration of 100 µM. For Hsp90 inhibition experiments, a final concentration of 0.5 µM GA or 0.8 µM 17-DMAG was added to CrFK-infected cells immediately after virus adsorption or at 1, 3 or 6 h post-infection (hpi). CQ was diluted in 1× PBS to a 50 mM working concentration and at a 100 µM final concentration in the autophagy inhibition experiments; CQ alone or in combination with GA was added to CrFK-infected cells immediately after viral adsorption. MG132 was diluted in DMSO and used at a 25 µM final concentration. MG132 was added to CrFK cells 2 h before infection with the FCV URB strain; immediately before infection, the monolayer of CrFK cells was washed with pre-warmed 1× PBS, and the infection was allowed for 1 h; then, cells were washed again with 1× PBS, and MG132 alone or in combination with GA was added to the medium. The drug vehicles, DMSO for both GA and MG132 or PBS 1× in the case of CQ, were used as controls for each condition assayed.

### MTT assays

Cell viability was measured by the 3-(4,5-dimethylthiazol-2-yl)−2,5-diphenyltetrazolium bromide (MTT) (Thermo Fisher Scientific) assays as previously described [22]. Briefly, CrFK cells were grown in 96-well plates, and when the monolayer reached 80% confluency, they were treated with GA, 17-DMAG, CQ or MG132 alone or with the combination of GA/CQ or GA/MG132. DMSO or 1× PBS-treated cells were used as controls for each concentration and combination tested. For each drug, different concentrations were assessed. Cell viability was measured at 0.2, 0.3, 0.5, 0.8 and 0.9 µM GA in MEM supplemented with 2% FBS; cells were treated with the drug for 13 h before adding the MTT reagent. Cell viability was measured at 25, 50, 75, 100 and 150 μΜ CQ in MEM supplemented with 2% FBS; cells were treated for 6 h before adding the MTT reagent. Cell viability was measured at 10, 20, 25, 40 and 50 µM MG132 in MEM supplemented with 2% FBS, and the drug was added 8 h before adding the MTT reagent. When the combination of GA/CQ was evaluated, the treatment with both drugs at 0.5 and 100 µM, respectively, lasted 6 h prior to the addition of the MTT reagent; for the combination of GA/MG132, cells were treated for 2 h with 25 µM MG132 alone, and afterwards, medium was removed, and the combination of 0.5 µM GA and 25 µM MG132 was added and incubated for 6 h before adding the MTT reagent. The number of viable cells was expressed as a percentage, normalized to the control non-treated cells [22,23].

### Western blot assays

Monolayers of mock-infected or infected CrFK cells were collected with a scrapper and, along with the supernatants, centrifuged at 1643 ***g*** for 5 min at 4 °C; the pellet was washed with PBS and centrifuged in the same conditions. Cells were lysed with RIPA buffer [150 mM sodium chloride, 1% Nonidet N-P40 (v/v), 0.5% deoxycholate (v/v), 0.1% SDS w/v and 50 mM Tris (pH 7.4) (J.T.Backer)] [24]. To the lysis buffer, 7× cOmplete Mini EDTA-free protease inhibitor cocktail, 200 mM PMSF and 0.5 M EDTA were added. Protein concentration in each sample was determined using Pierce BCA Protein Assay Kit (Thermo Scientific), and 10 µg of total protein extracts was heated at 80 °C for 5 min, analysed by SDS-PAGE and transferred to a 0.22 µM nitrocellulose membrane. Membranes were blocked at room temperature (RT) for 30 min with 5% skimmed milk in 1× PBS −1% Tween 20 for antibodies raised in mice and most antibodies raised in rabbits. For anti-LC serum, membranes were blocked in 1× PBS −1% Triton-X100 at 37 °C for 30 min. In all experiments, 1× PBS −1% Tween 20 was used to dilute anti-mouse antibodies, while 1× PBS −1% Triton-X100 was used to dilute anti-rabbit antibodies. In all cases, primary antibodies were incubated at 4 °C, overnight. Then, membranes were washed with either 1× PBS −1% Tween or 1× PBS-1%-Triton-X100, and secondary antibodies were incubated at RT for 2 h and developed using chemiluminescence (Pierce SuperSignal West Femto), detected using autoradiography film (Carestream film). Protein levels were quantified by scanning the resulting films and measuring band intensities with FIJI software (https://fiji.sc).

### Antibodies for Western blot assays

Primary antibodies are anti-Hsp90β (1 : 12 000, ab29270; Abcam), anti-Hsp70 (1 : 1 000, sc-66 048; Santa Cruz Biotechnology), anti-LC3β (1 : 4 000, sc-271 625; Santa Cruz Biotechnology), anti-Annexin A2 (1 : 1 000 000, sc-48 397; Santa Cruz Biotechnology), anti-β-Actin (1 : 40  000, sc47 778; Santa Cruz Biotechnology) and anti-FCV (sc-80 785; Santa Cruz Biotechnology). Sera against viral proteins, anti-NS3 1 : 40 000, anti-VP2 1 : 500 and anti-NS6/7 1 : 12 000, were kindly donated by Ian Goodfellow, University of Cambridge, and anti-LC 1 : 60  000 and anti-VP1 1 : 90 000 were developed by our workgroup [25]. Secondary antibodies are anti-mouse (1 : 20 000, AB_10 015 289; Jackson ImmunoResearch) and anti-rabbit (1 :  20 000, AB_2 313 567; Jackson ImmunoResearch).

### Immunofluorescence assays

CrFK cells were grown on coverslips and mock-infected or infected with FCV URB strain at an MOI of 5, GA- or DMSO-treated; infection was allowed for 6 h, and cells were washed two times with prewarmed and filtered 1× PBS for 5 min. After washing, cells were fixed with 4% formaldehyde in 1× PBS at RT for 15 min, washed three times with filtered 1× PBS, permeabilized with 0.001% Triton X-100 in 1× PBS for 5 min, washed three more times with filtered 1× PBS and blocked with 0.5 % porcine skin gelatin (Sigma-Aldrich) in filtered PBS at RT for 30 min. Primary antibodies were used as follows: anti-Hsp90β (1:100, ab29270; Abcam) or anti-VP1 serum (1:200, own laboratory); both antibodies were diluted in filtered PBS and incubated overnight at 4 °C. Then, samples were washed three times with filtered 1× PBS for 5 min before incubating with the corresponding secondary antibodies and diluted 1:200 in 1× PBS at RT for 2 h. Samples were washed thrice with filtered 1× PBS and incubated with DAPI at a 1 mg ml^−1^ concentration at RT for 5 min. Samples were washed thrice with filtered 1× PBS, mounted with VECTASHIELD (Vector Laboratories), and analysed with a Zeiss LSM-700 confocal microscope.

### Proximity ligation assays (Duo-link)

The interaction between Hsp90 and FCV’s VP1 was detected by proximity ligation assays (PLA) using the Duo-link *In Situ*-Fluorescence Kit (Sigma-Aldrich) in FCV URB-infected cells. The assay was performed according to the manufacturer’s instructions; briefly, cells grown in coverslips were infected at an MOI of 5 and, after 6 h, fixed and permeabilized as described above. After treatment with the blocking agent at RT for 30 min, samples were incubated with primary antibodies overnight at 4 °C temperature as follows: anti-Hsp90β (1:100) and anti-FCV (1:300), both diluted in the antibody’s dilution agent. Mock-infected cells incubated with both primary and secondary antibodies, and FCV-infected cells incubated only with the anti-Hsp90β antibody were used as negative controls.

### Statistical analysis

Statistical analysis was performed using the GraphPad Prism 10 software (CA, USA). Comparisons were made using t-tests between samples and their corresponding control. Error bars represent the sd from three independent experiments unless stated otherwise.

## Results

### Hsp90 levels did not change in FCV-infected cells

**Fig. 1.**
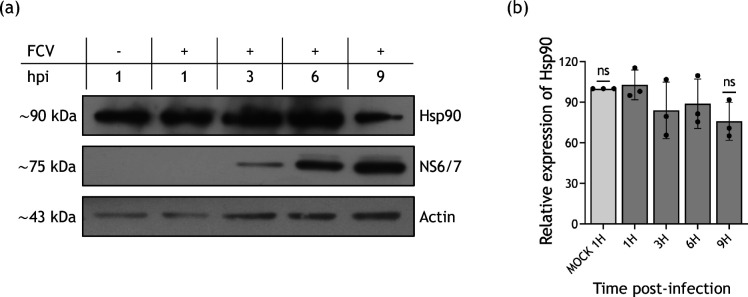
Hsp90 levels do not change during FCV infection. Total protein extracts from mock-infected and FCV-infected cells at 1, 3, 6 and 9 hpi were obtained, and (**a**) Hsp90 and NS6/7 protein levels were analysed by Western blotting; a representative image of three independent experiments is shown. Actin was used as a loading control. (**b**) Relative expression levels of Hsp90 from three independent experiments. Densitometric values were obtained using FIJI software, and the statistical analysis of the means was analysed with GraphPad Prism 10 software by two-way ANOVA. No significant (ns) differences are indicated.

Hsp90 is one of the most studied chaperones in the context of viral infections. Its relevance for achieving an efficient replication cycle in other members of the family *Caliciviridae*, such as the murine and human noroviruses, has been reported [15]; however, its importance for FCV replication is still unknown. For this purpose, the levels of Hsp90 during the FCV replication cycle were evaluated. Total protein extracts from mock-infected and infected cells at 1, 3, 6 and 9 h were subjected to SDS-PAGE, and Hsp90 protein levels were evaluated by Western blotting (Fig. 1a and b). Similar levels of Hsp90 were observed in both mock-infected and infected cells at all analysed times, suggesting that its levels remain stable during FCV infection.

### Hsp90 activity is essential for efficient FCV particle production

**Fig. 2.**
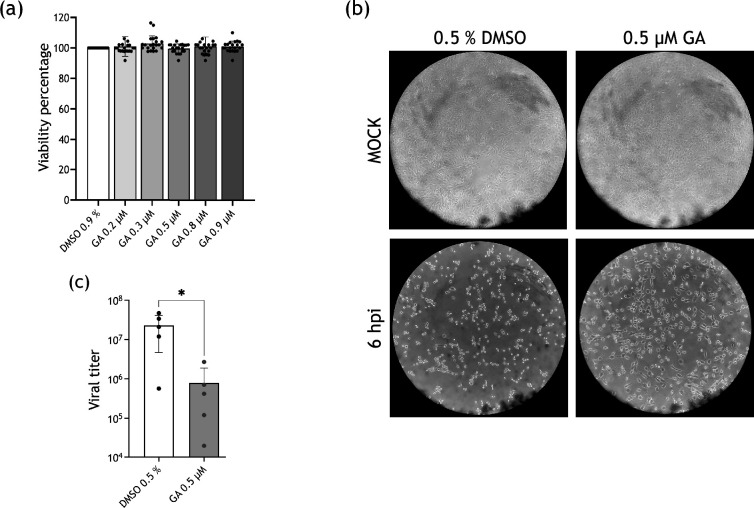
Hsp90 is required for efficient viral particle production during FCV infection. (**a**) CrFK cells were treated with 0.2, 0.3, 0.5, 0.8 and 0.9 μΜ GA, and cell viability was assessed by an MTT assay. (**b**) Mock-infected (upper panel) and FCV-infected cells at an MOI of 5 (lower panel) were treated with 0.5% DMSO or 0.5 µM GA and analysed by optical microscopy. (**c**) Supernatants from FCV-infected cells treated with 0.5% DMSO or 0.5 µM GA were obtained, and viral titre was quantified by plaque assays. Statistical analysis was performed using GraphPad Prism 10 software. Values of *P*<0.05 (*), calculated by t-test, are indicated.

Once it was determined that Hsp90 levels do not vary during FCV infection, the consequences of its inhibition in FCV replication were assessed by treating cells with GA, a specific inhibitor [26] that binds the ATPase active site of Hsp90 [27]. Previously, an MTT assay was used to evaluate viability in cells treated with GA at different concentrations (Fig. 2a).

None of the assayed GA concentrations (0.2, 0.3, 0.5, 0.8 and 0.9 µM) affected CrFK cell viability (Fig. 2a); therefore, 0.5 µM GA concentration was used in all further experiments. To determine if Hsp90’s activity was required for FCV replication, CrFK cells were infected at an MOI of 5 and treated with either GA or the drug vehicle (DMSO) immediately after 1 h of viral adsorption. After 6 hpi, supernatants were collected, and the viral particle production associated was quantified by plaque assay (Fig. 2c). A reduction of 1.5 logs in viral titre from cells treated with GA when compared with the vehicle was observed, strongly suggesting that the FCV requires Hsp90 in its active form to accomplish an efficient replication. This result correlates with a delayed onset of the cytopathic effect observed by optical microscopy during the experiment in treated cells compared with controls (Fig. 2b).

### Hampering Hsp90 activity reduces FCV’s VP1 but not NS6/7, NS3 and VP2 protein levels

**Fig. 3.**
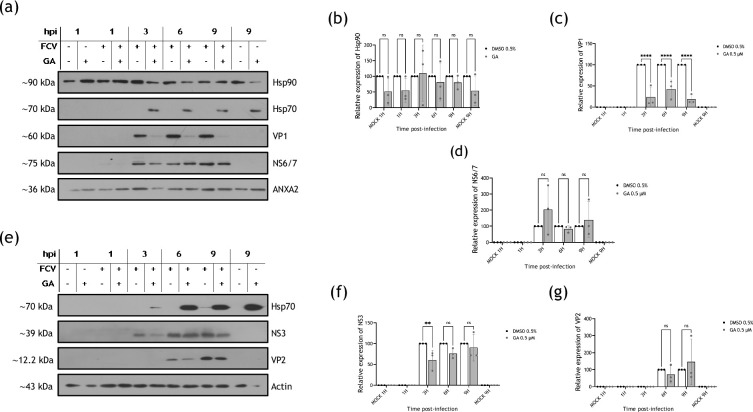
Hsp90 inactivation by geldanamycin causes a reduction in VP1 but not in NS6/7 NS3 and VP2 protein levels. Mock-infected and FCV-infected cells at an MOI of 5 were treated with 0.5% DMSO or 0.5 µM GA. Total protein extracts were obtained, and the levels of (**a**) Hsp90, VP1, NS6/7, NS3 and VP2 were analysed at 3, 6 and 9 hpi by Western blotting. A representative blot of three independent experiments is shown. Hsp70 was used as a control of Hsp90 inhibition and annexin A2 (ANXA2) and actin as loading controls. Mock-infected treated and non-treated cells were included as controls. The relative expression of (**b**) Hsp90, (**c**) VP1, (**d**) NS6/7, (**f**) NS3 and (**g**) VP2 from three independent experiments is shown. Densitometric values were obtained using FIJI software, and the statistical analysis was performed using GraphPad Prism 10 software. No significant (ns) differences are indicated. Values of *P*<0.01 (**) and *P*<0.0001 (****) calculated by two-way ANOVA are indicated.

To better comprehend the Hsp90 role in FCV infection, cells were treated with GA, and viral protein levels were evaluated throughout the replication cycle. Since both polymerases and structural proteins are the most common Hsp90 client proteins of viral origin, the levels of both the protease-polymerase NS6/7 and the capsid protein VP1 were assessed by Western blotting (Fig. 3). No significant reduction in the NS6/7 protein levels at 3, 6 and 9 hpi was detected from cells treated with the Hsp90 inhibitor, in comparison to the NS6/7 levels from the drug vehicle-treated cells (Fig. 3a and d). However, a significant reduction in the VP1 levels at 3, 6 and 9 hpi was observed in cells treated with GA compared with those treated with DMSO (Fig. 3a and c). To determine if other viral proteins such as the non-structural protein NS3 and the structural protein VP2 were affected when Hsp90 was inhibited, their level in the presence of GA was also assessed by Western blotting (Fig. 3e). No significant reduction in both protein levels at 3, 6 and 9 hpi was detected (Fig. 3e, f and g). Hsp70 was used as a positive control of Hsp90 inhibition. The marked reduction in VP1 protein levels without Hsp90 activity correlates with the delayed onset of the cytopathic effect and the viral titre decrease shown in Fig. 2, suggesting that Hsp90 is required for VP1 stability.

**Fig. 4.**
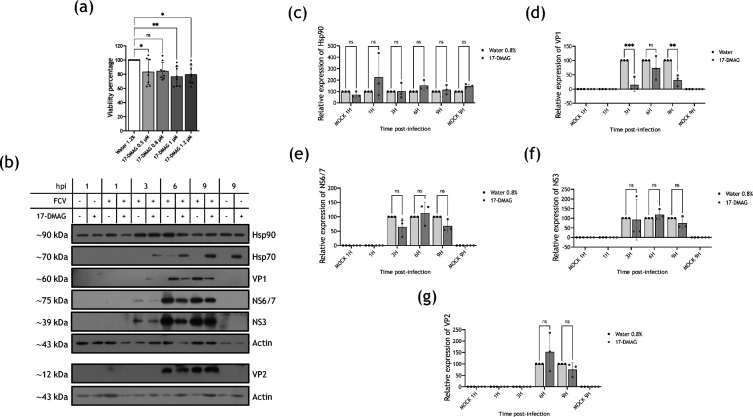
Hsp90 inactivation by 17-DMAG causes a reduction in VP1 but not in NS6/7, NS3 and VP2 protein levels. (**a**) CrFK cells were treated with 0.5, 0.8, 1.0 and 1.2 μΜ 17-DMAG, and cell viability was assessed by an MTT assay. Mock-infected and FCV-infected cells at an MOI of 5 were treated with the vehicle or 0.8 µM 17-DMAG. Total protein extracts were obtained, and the levels of (**b**) Hsp90, (**c**) VP1, (**d**) NS6/7, (**e**) NS3 and (**f**) VP2 were analysed at 3, 6 and 9 hpi by Western blotting. A representative blot of three independent experiments is shown. VP2 protein expression was analysed in a gel run in parallel. Hsp70 was used as a control of Hsp90 inhibition and actin as a loading control. The relative expression of (**c**) Hsp90, (**d**) VP1, (**e**) NS6/7, (**f**) NS3 and (**g**) VP2 from three independent experiments is shown. Densitometric values were obtained using FIJI software, and the statistical analysis was performed using GraphPad Prism 10 software. No significant (ns) differences are indicated. Values of *P*<0.05 (*) and *P*<0.01 (**) calculated by two-way ANOVA are indicated.

Another potent and soluble Hsp90 inhibitor used in preclinical studies that involve Hsp90 as a therapeutic target for cancer treatment is the semi-synthetic GA derivative 17-dimethylaminoethylamino-17-demethoxygeldanamycin (17-DMAG) that binds to Hsp90 and inhibits its function but has the advantage to be water soluble and has a good bioavailability [28]. Thus, this drug was used as another approach to determine the effect of Hsp90 inhibition in FCV replication. MTT assay to evaluate the viability of CrFK cells treated with 0.5, 0.8, 1.0 and 1.2 µM 17-DMAG was performed (Fig. 4a). Both 0.5 and 0.8 µM 17-DMAG treatment resulted in an 80% of cell viability; thus, 0.8 µM concentration was used in all further experiments. CrFK cells were infected at an MOI of 5 and treated with either 17-DMAG or its vehicle (water) immediately after 1 h of viral adsorption, and viral protein levels were evaluated throughout the replication cycle (Fig. 4b). When using GA, no significant reduction in the NS6/7 and VP2 protein levels at 3, 6 and 9 hpi and NS3 protein levels at 6 and 9 hpi was detected from cells treated with the Hsp90 inhibitor, in comparison to these protein levels from the vehicle-treated cells (Fig. 4b, e, f and g, respectively). However, a reduction in the VP1 levels at 3, 6, and 9 hpi that was significant at 3 and 9 hpi was observed (Fig. 4b and d); results that are in accordance with the ones obtained with GA and support the suggestion that Hsp90 is required for VP1 production and/or stability.

### The lack of activity of Hsp90 does not impair the translation of the FCV’s subgenomic RNA

The reduction in VP1 due to the absence of Hsp90 activity could result from its degradation or impairment in the synthesis or translation of the RNA template from which this protein is translated. While NS6/7 is encoded and translated from the genomic RNA, VP1 is not. Despite being encoded in the genomic RNA, VP1 is translated from a subgenomic RNA [29]; therefore, in infected cells, NS6/7 and VP1 proteins come from different RNA templates.

The protein VP1 is initially translated from the subgenomic RNA as a precursor (LC–VP1) late in infection and is further cleaved by the NS6/7 viral proteinase to produce the LC and VP1 mature proteins [30]; therefore, the absence or presence of the precursor protein would shed light on whether Hsp90’s activity has an impact on sgRNA translation.

**Fig. 5.**
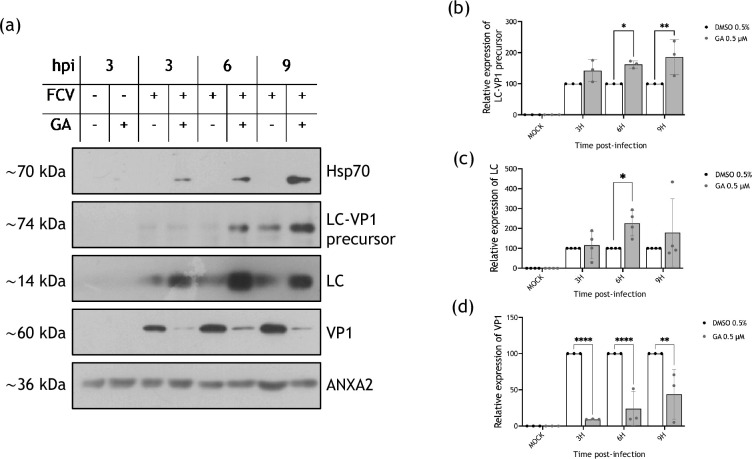
The hampering of Hsp90 activity does not affect the LC–VP1 precursor protein production. Mock-infected and FCV-infected cells at an MOI of 5 were treated with 0.5% DMSO or 0.5 µM GA for 3, 6 and 9 hpi. (**a**) Total protein extracts were obtained, and the presence or LC–VP1 precursor, LC and VP1 levels were analysed by Western blotting. Representative blot of three independent experiments is shown. Hsp70 was used as a control of Hsp90 inhibition and annexin A2 (ANXA2) as a loading control. Mock-infected treated and non-treated cells were included as controls. The relative expression of (**b**) LC–VP1 precursor, (**c**) LC and (**d**) VP1 from three independent experiments is shown. Densitometric values were obtained using FIJI software, and the statistical analysis of the means was analysed with GraphPad Prism 10 software. No significant (ns) differences are indicated. Values of *P*<0.05 (*), *P*<0.01 (**) and *P*<0.0001 (****), calculated by two-way ANOVA, are indicated.

To detect the LC–VP1 precursor, total protein extracts from infected cells treated with DMSO or GA were obtained at different hpi. The LC–VP1 precursor, as well as the mature forms of LC and VP1 proteins, were detected by Western blotting (Fig. 5); an anti-LC serum was used to detect both LC (14 kDa) and the LC–VP1 precursor (74 kDa) (Fig. 5), while the anti-VP1 was used to detect both VP1 and LC–VP1.

As shown in Fig. 4, the LC–VP1 precursor was detected by Western blotting from 3 and up to 9 hpi in all conditions (Fig. 5a). An augment in the protein levels from cells treated with the Hsp90 inhibitor was evident in all times post-infection tested and resulted significant at 9 hpi (Figs5a  4b). In concordance with the detected augment of the LC–VP1 precursor, cells treated with GA also showed higher levels of the mature form of the LC protein in comparison to the DMSO-treated cells at 6 hpi (Fig. 5c), suggesting that the sgRNA synthesis and translation are not hindered in the absence of Hsp90 activity. Therefore, the reduction in the VP1 protein levels in the presence of GA indicates that this viral protein requires Hsp90 activity for its stability rather than for its production, strongly suggesting that VP1 is a client protein of this cellular chaperone.

### The FCV’s VP1 is a client protein of Hsp90

**Fig. 6.**
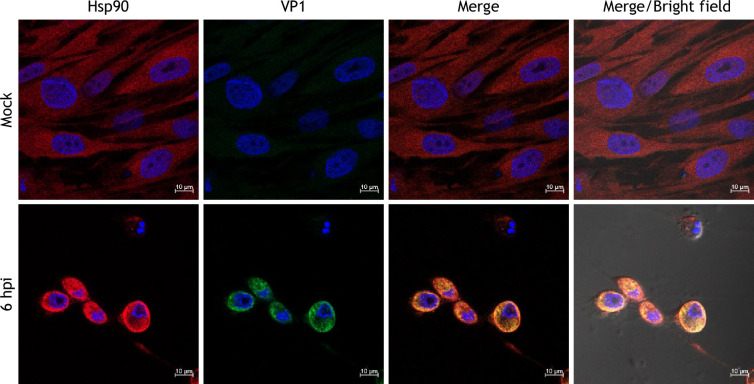
Hsp90 and VP1 colocalize during FCV infection. Mock-infected and FCV-infected cells at an MOI of 5, for 6 h, grown on coverslips were immunostained with anti-Hsp90 (red) and VP1 (green) antibodies, followed by Alexa Fluor 594 (red) and 488 (green) as secondary antibodies, respectively. Images correspond to a z-stack of 15 slices and are representative of three independent experiments. Cells were examined in a Zeiss LSM 700 confocal microscope.

All results obtained pointed out the need for Hsp90 activity to warrant an efficient FCV replication cycle, since the use of the specific inhibitor GA delayed the onset of the cytopathic effect and resulted in a substantial decrease in viral particle production. This is most probably due to the reduction in VP1 protein levels, since neither NS6/7 nor LC protein levels were reduced. All collected data strongly suggest that Hsp90 is the molecular chaperone of VP1. To confirm that VP1 is a client of Hsp90, it was necessary to demonstrate that both proteins interact. To determine their possible interaction, both an immunofluorescence and a PLA were performed using confocal microscopy (Fig. 6).

**Fig. 7.**
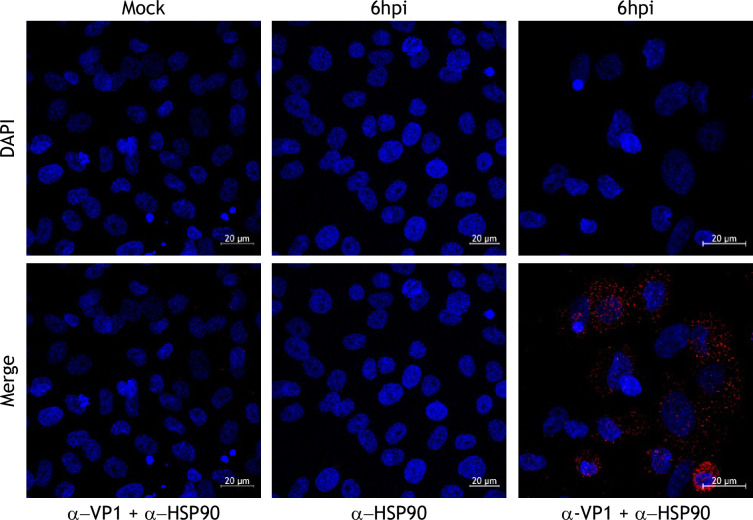
Hsp90 and VP1 interact during FCV infection. Proximity ligation assay (PLA-Duo-link) between VP1 and Hsp90 in FCV-infected cells at an MOI of 5, for 6 hpi. PLA signal (red) represents dual recognition against VP1 and Hsp90. DAPI was used for nuclear (blue) staining. Cells were examined in a Zeiss LSM 700 confocal microscope.

A colocalization between Hsp90 and VP1 proteins was observed in the cytoplasmic region from infected cells with a mean Pearson’s colocalization coefficient of 0.7, suggesting the proximity of both molecules (Fig. 6). To confirm the direct interaction between both proteins, a PLA analysis was performed in FCV-infected CrFK cells. A positive signal was observed as red fluorescent dots in the cytoplasm of infected cells at 6 hpi when antibodies against both Hsp90 and VP1 were used, strongly suggesting the interaction of both molecules (Fig. 7). No signal was detected in the mock-infected cells incubated with both anti-Hsp90 and anti-VP1 antibodies or with the anti-VP1 antibody alone, demonstrating the specificity of the signal (Fig. 7). These results confirm that VP1 and Hsp90 are interacting in FCV-infected cells, which supports the notion that VP1 is a client protein of Hsp90 chaperone.

### The autophagy pathway is not involved in VP1 degradation during FCV infection

Once it was established that VP1 is a client protein of Hsp90 and that its lack of activity results in the reduction in VP1 levels, it was important to identify its degradation route. The two main degradation pathways in eukaryotic cells are autophagy and the ubiquitin–proteasome [31].

Autophagy’s involvement was evaluated with chloroquine (CQ), which has been reported to hinder autophagy by inhibiting autophagosome–lysosome fusion, as well as lysosome acidification, the prevalence of which might vary depending on the assessed system [32,33].

**Fig. 8.**
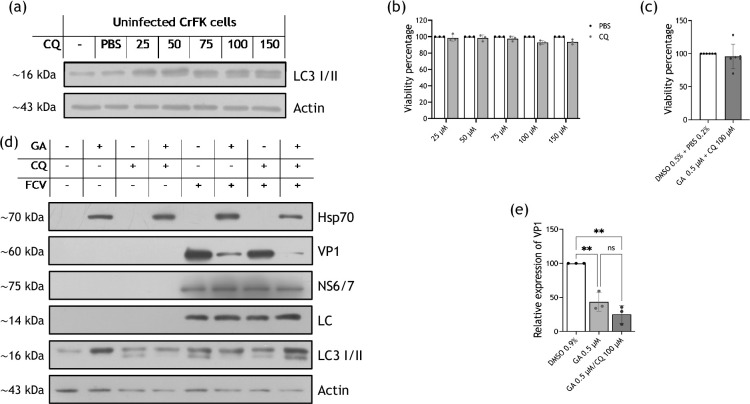
Autophagy is not involved in VP1 degradation when Hsp90 is inactive. (**a**) Total protein extracts from CrFK cells treated with 1.5% PBS 1× or CQ at 25, 50, 75, 100 and 150 µM were obtained, and the LC3 II protein accumulation was analysed by Western blotting; a representative image of two independent experiments is shown. The viability of CrFK cells treated with (**b**) CQ at 25, 50, 75, 100 and 150 µM or the corresponding concentration of 1× PBS or (**c**) the combination of 0.5 µM GA and 100 µM CQ or 0.5% DMSO and 0.2% 1× PBS was assessed by an MTT assay; the mean of three independent experiments is shown. (**d**) Mock-infected or FCV-infected cells at an MOI of 5, for 6 h, were treated with 0.5% DMSO or 0.5 µM GA, and 0.2% PBS 1×, or 100 µM CQ, or the combination of both drugs. Total protein extracts were obtained, and the presence of VP1, NS6/7 and LC proteins was analysed by Western blotting. Hsp70 was used as a control of Hsp90 inhibition, LC3I/II as a control of autophagy inhibition and actin as a loading control. The relative expression of (**e**) VP1 from two independent experiments is shown. Densitometric values were obtained using FIJI software, and the statistical analysis of the means was analysed with GraphPad Prism 10 software. Values of *P*<0.01 (**) calculated by two-way ANOVA are indicated.

The CQ inhibitory concentration of autophagy in CrFK cells was determined using the drug at 25, 50, 75, 100 and 150 µM and measuring the accumulation of LC3 II protein since the conversion from LC3I to LC3II is widely used to monitor autophagy [34,35] (Fig. 8a). Moreover, cell viability in the presence of different concentrations of CQ was determined by an MTT assay (Fig. 8b). The CQ concentration chosen for autophagy inhibition was 100 µM, due to the effect on LC3II accumulation and to the lack of a negative effect on CrFK cell viability alone or with 0.5 µM GA (Fig. 8c).

To determine if VP1 protein was degraded through the autophagy pathway when Hsp90 is inactive, cells were treated with GA, GA and CQ or the drug vehicles (Fig. 8d), and the levels of VP1, NS6/7 and LC were determined. As expected, no changes in NS6/7 or LC protein levels were observed in cells treated with 0.5 µM GA (as previously shown, 100 µM CQ alone or the combination of both 0.5 µM GA and 100 µM CQ) (Fig. 8d). However, when the autophagy pathway in GA-treated samples was inhibited, VP1 basal levels could not be restored (Fig. 8d and e), strongly suggesting that autophagy is not its degradation route when HSP90 is inactive. Again, the presence of Hsp70 was used as a positive control of Hsp90 inhibition, while the LC 3-II accumulation is the positive control of autophagy inhibition. The attenuation of the autophagic process inferred by the lack of LC3II accumulation in cells treated with 0.5 µM GA alone correlates with previous reports that Hsp90 inhibition hinders the autophagy, probably by the destabilization of ATG7 [36,37], and also impairs the accumulation of LC3 [38]. Taken together, all these results indicate that autophagy is not the pathway implicated in the degradation of VP1 when Hsp90 is inactive.

### The proteasome activity is needed to achieve an efficient replication of the FCV

To evaluate the ubiquitin–proteasome pathway as the degradation route of VP1, MG132, a synthetic aldehyde that, depending on the concentration range, binds specifically in a reversible way to the proteasome β5 catalytic subunit, but can also bind to the β1 and β2 subunits at higher concentrations, was used [39,40].

**Fig. 9.**
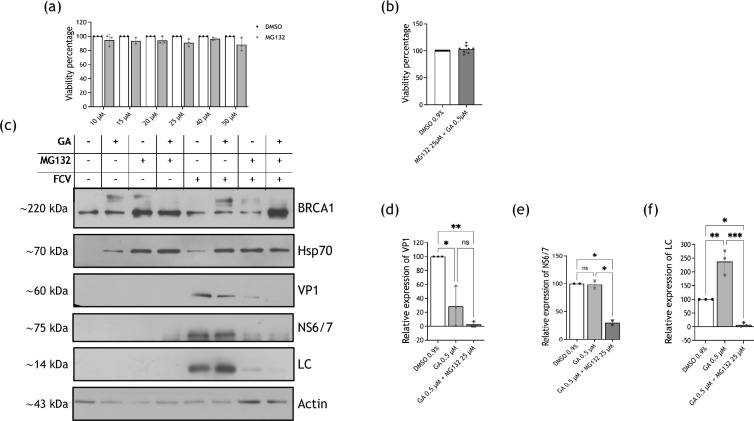
An active proteasome is required for an FCV efficient replication. CrFK cells were treated with (**a**) MG132 at 10, 15, 20, 25, 40 and 50 µM or the corresponding concentrations of DMSO or (**b**) with the combination of 25 mM MG132 and 0.5 µM GA or 0.9% DMSO, and cell viability was evaluated by the MTT assay; the mean of three independent experiments is shown. (**c**) Mock-infected or FCV-infected cells at an MOI of 5, for 6 hpi, were treated with 0.5 µM GA, 25 µM MG132, the combination of both drugs or the corresponding concentration of DMSO, and the levels of VP1, NS6/7 and LC proteins were analysed by Western blotting. Hsp70 was used as a control of Hsp90 inhibition, BRCA1 as a control of proteasome inhibition and actin as a loading control. The relative expression of (**d**) VP1, (**e**) NS6/7 and (**f**) LC from two independent experiments is shown. Densitometric values were obtained using FIJI software, and the statistical analysis of the means was analysed with GraphPad Prism 10 software. No significant (ns) differences are indicated. Values of *P*<0.05 (*), *P*<0.01 (**) and *P*<0.001 (***), calculated by two-way ANOVA, are indicated.

CrFK cells were treated with 10, 15, 20, 25, 40 and 50 µM MG132, and an MTT assay was performed to determine the effect of proteasome inhibition on cell viability. (Fig. 9a). None of the MG132-assayed concentrations significantly affected CrFK cell viability; therefore, 25 µM was the MG132 concentration used in all subsequent experiments. The combination of both 0.5 µM GA and 25 µM MG132 was assessed by an MTT assay, which showed no significant reduction in cell viability (Fig. 9b).

To determine if VP1 is degraded via proteasome when Hp90 is inactive, both inhibitors GA and MG132 were added to FCV-infected cells after virus adsorption. Controls included non-infected and infected cells treated with DMSO, 0.5 µM GA, 25 µM MG132 or both drugs combined (Fig. 9c). As in previous experiments, the relative expression of VP1, NS6/7 and LC were assessed by Western blotting. All NS6/7, LC and VP1 proteins showed the previously observed behaviour when inhibiting Hsp90 with 0.5 µM GA; however, when inhibiting the proteasome by the addition of MG132 or when adding both drugs, a significant reduction in the relative expression levels of NS6/7 and LC was observed (Fig. 8c and e). In the case of VP1, adding GA and MG132 resulted in an even greater reduction in protein levels than in the presence of GA alone (Fig. 9c and d). Thus, VP1 levels in GA-treated cells could not be rescue by proteasome inhibition. Hsp70 was used as a positive control of the Hsp90 and proteasome activity inhibition since hindering proteasome activity triggers the so-called heat shock response that consists in the immediate synthesis of cytoprotective proteins, which includes Hsp70 [41,42]. The breast cancer 1 protein (BRCA1), which is degraded in a proteasome-dependent manner, was used as a positive control of proteasomal inhibition [43]. Taken together, these results suggest that proteasome activity is required during the FCV replication cycle.

## Discussion

Viruses have developed several strategies to hijack cellular pathways and factors, such as molecular chaperones, to achieve an efficient replication or evade immune responses. The Hsp90 chaperone family is one of the most studied in the context of viral infections; nevertheless, its importance during the replication cycle of FCV has not been studied, although its relevance for efficient replication of other members of the family *Caliciviridae*, such as MNV and HuNoV, has been previously reported [15].

Although we found that Hsp90 is not modulated during FCV infection, its inhibition with GA, a specific inhibitor of its ATPase activity, strongly reduced viral particle production, suggesting an important role of this molecular chaperone in the FCV replication cycle.

Even though both polymerases and structural proteins are common clients of the Hsp90 system [14], VP1 but not the NS6/7 relative expression levels were reduced when inhibiting this chaperone’s activity either by GA or by 17-DMAG. Moreover, the levels of the non-structural protein NS3 and structural protein VP2 were not modified during Hsp90 inhibition. Since VP1 is produced as a precursor protein that is further processed into the mature LC and VP1 proteins, detecting either the precursor or the mature LC levels helped us to determine if the reduction in the VP1 levels was due to changes in its synthesis or stability. Our results demonstrate that levels of both the LC–VP1 precursor and LC protein remained unchanged when Hsp90 is inactive, strongly suggesting that VP1 requires the activity of Hsp90 for its stability rather than its synthesis, indicating that VP1 is a client protein of Hsp90. Additionally, the fact that VP2 levels were not altered upon inhibition of Hsp90 activity further supports the conclusion that the reduction in VP1 levels is due to changes in its stability, as VP2 relies on VP1 for its translation [44,45]. The degradation of VP1 when Hsp90 was inactive and the interaction found between both proteins confirmed that VP1 is a client of Hsp90, as reported previously for MNV VP1 [15]. This chaperone might regulate the stability of the capsid protein at the post-translational level, most probably to facilitate the proper capsid assembly, as reported for other viruses [46,47].

The presence of LC–VP1 precursor and LC when Hsp90 was inhibited indicates that none of these two proteins are clients of this chaperon; furthermore, the fact that the levels of both proteins tend to increase in all the times analysed suggests that Hsp90 regulates the replication or translation of the subgenomic RNA. In this regard, this chaperon protein interacts with the 3′ UTR of the Bamboo mosaic virus RNA and facilitates its replication initiation [48]. The interaction of Hsp90 with the ends of the FCV RNAs has not been reported yet; however, it binds to both ends of the MNV genomic RNA [49], suggesting that this protein could be participating in the RNA replication and/or translation of caliciviruses, as other cellular proteins.

To identify the degradation route of VP1 in the absence of Hsp90 activity, both autophagy and proteasome routes were studied. Autophagy inhibition by CQ did not restore VP1 basal levels in GA-treated cells, indicating that this pathway is not involved in VP1 degradation when Hsp90 is inactive. Moreover, CQ treatment had no detrimental effect on the relative expression of any of the viral proteins tested at the time points assessed. Our results showed that autophagy is triggered during FCV infection, as demonstrated by the induction of LC3II and in concordance with a very recent report that indicates that autophagy is induced as a mechanism to attenuate the cell’s antiviral immune response [50]. On the other hand, the attenuation of the autophagic process inferred by the lack of LC3II accumulation in cells treated with 0.5 µM GA alone correlates with previous reports that Hsp90 inhibition hinders the autophagy, probably by the destabilization of ATG7 [36,37], and that also impairs the accumulation of LC3II [38].

On the other hand, inhibiting the proteasome activity by MG132 did not restore the VP1 levels in GA-treated cells either, suggesting that this pathway was not the VP1 degradation route.

However, the significant reduction in the three assessed viral proteins NS6/7, LC and VP1 in the presence of MG132 indicates that an active proteasome is required, at least in the early/middle steps of FCV replication. These results evidence for the first time the need for an active proteasome for FCV replication; yet, it was not possible to determine if this pathway is involved in VP1 degradation when Hsp90 is inactive. Further experiments such as the expression of FCV VP1 in a virus-free system, in the presence of GA, might be required to identify the role of proteasome in VP1 degradation.

Since the proteasome system regulates fundamental cellular functions through ensuring protein quality control and maintaining critical levels of regulatory proteins [51], it is not surprising that viruses control this pathway to favour viral replication and propagation and combat the host anti-viral machinery. The need for an active proteasome has been reported to have different roles for several viruses [52,53], for example, in the production of the genomic and subgenomic RNAs in astrovirus infection [54]. On the other hand, proteasomes can be utilized by positive-sense RNA viruses to regulate their life cycles by eliminating excess viral proteins that prevent viral replication, for example, to maintain the appropriate balance of viral proteins such as replicases and proteases [55,56] and to escape host immune surveillance [57]. Another possibility is that proteasome could be degrading cellular factors that impair viruses to regulate their life cycles.

In this sense, the increased levels of LC–VP1 precursor and LC when Hsp90 is inactive could be explained by the presence of a cellular protein, client of Hsp90, with a restricting role of the subgenomic RNA replication or translation and whose proteasomal-mediated degradation is promoted during infection. Thus, the proteasome could be playing a role at different levels in the replication cycle of the FCV.

## Conclusion

Here, we demonstrated the need for Hsp90 activity for efficient replication of FCV and identified VP1 as a client protein of Hsp90, identifying a potential therapeutic target for the treatment of FCV infection as previously suggested for MNV and HuNoV. Moreover, we also found the induction of autophagy during FCV infection, as recently reported by Mao *et al*., and ruled out this pathway in VP1 degradation. Finally, we reported for the first time the requirement for an active proteasome during FCV infection, leaving an open door to redirect the strategy to further determine if this pathway is responsible for the VP1 degradation route when Hsp90 is inhibited.

## Abbreviations

CQ: chloroquine
CrFK: Crandell–Reese feline kidney
FCV: Feline calicivirus
GA: geldanamycin
hpi: hour post-infection
Hsp90: heat shock protein 90
HuNoV: human norovirus
LC: leader of the capsid
MEM: Minimal Essential Medium
MG132: carbobenzoxy-l-Leucyl-l-Leucyl-l-Leucinal
MNV: murine norovirus
MOI: multiplicity of infection
MTT: 3-(4,5-dimethylthiazol-2-yl)−2,5-diphenyltetrazolium bromide
PLA: proximity ligation assays
RT: room temperature
sgRNA: subgenomic RNA
URB: Urbana
VP1: viral protein 1
VP2: viral protein 2
